# Clinical significance of pneumatosis intestinalis – correlation of MDCT-findings with treatment and outcome

**DOI:** 10.1007/s00330-016-4348-9

**Published:** 2016-04-22

**Authors:** Marc-Olivier Treyaud, Rafael Duran, Marc Zins, Jean-Francois Knebel, Reto A. Meuli, Sabine Schmidt

**Affiliations:** 1 Department of Diagnostic and Interventional Radiology, Lausanne University Hospital, Rue du Bugnon 46, 1011 Lausanne, Switzerland; 2 Department of Radiology, Fondation Hôpital St Joseph, 185 Rue Raymond Losserand, 75014 Paris, France

**Keywords:** Pneumatosis intestinalis, Multidetector computed-tomography, Intestinal ischemia, Portomesenteric venous gas, Intestines

## Abstract

**Objectives:**

To evaluate the clinical significance of pneumatosis intestinalis (PI) including the influence on treatment and outcome.

**Method and Materials:**

Two radiologists jointly reviewed MDCT-examinations of 149 consecutive emergency patients (53 women, mean age 64, range 21-95) with PI of the stomach (*n* = 4), small (*n* = 68) and/or large bowel (*n* = 96). PI extension, distribution and possibly associated porto-mesenteric venous gas (PMVG) were correlated with other MDCT-findings, risk factors, clinical management, laboratory, histopathology, final diagnosis and outcome.

**Results:**

The most frequent cause of PI was intestinal ischemia (*n* = 80,53.7 %), followed by infection (*n* = 18,12.1 %), obstructive (*n* = 12,8.1 %) and non-obstructive (*n* = 10,6.7 %) bowel dilatation, unknown aetiologies (*n* = 8,5.4 %), drugs (*n* = 8,5.4 %), inflammation (*n* = 7,4.7 %), and others (*n* = 6,4 %). Neither PI distribution nor extension significantly correlated with underlying ischemia. Overall mortality was 41.6 % (*n* = 62), mostly related to intestinal ischemia (*p* = 0.003). Associated PMVG significantly correlated with underlying ischemia (*p* = 0.009), as did the anatomical distribution of PMVG (*p* = 0.015). Decreased mural contrast-enhancement was the only other MDCT-feature significantly associated with ischemia (*p p* < 0.001). Elevated white blood count significantly correlated with ischemia (*p* = 0.03).

**Conclusion:**

In emergency patients, ischemia remains the most common aetiology of PI, showing the highest mortality. PI with associated PMVG is an alerting sign. PI together with decreased mural contrast-enhancement indicates underlying ischemia.

**Key Points:**

• *In emergency patients, PI may be caused by various disorders*.

• *Intestinal ischemia remains the most common cause of PI in acute situations*.

• *PI associated with decreased mural contrast-enhancement indicates acute intestinal ischemia*.

• *PI associated with PMVG should alert the radiologist to possible underlying ischemia*.

## Introduction

Pneumatosis intestinalis (PI) is defined as the presence of gas bubbles within the intestinal wall, mostly located in the mucosa or submucosa. PI may involve any part of the digestive tract. It can occur with gas in the portal or mesenteric veins [1, 2]. PI was first described as early as 1730 by Duvernoy during a cadaveric dissection [3], while portomesenteric venous gas (PMVG) was initially reported by Wolf and Evans in 1955 [4]. Today, PI and PMVG are mostly detected on cross-sectional imaging modalities, in particular by multidetector computed-tomography (MDCT) in the emergency department. The detection of PI had historically been considered an alarming radiological feature, since it was almost always interpreted as acute bowel ischemia, thus necessitating immediate surgery [5, 6]. The patient’s situation is even more critical whenever PMVG is present [1, 2, 7].

However, PI is not a primary disease, but rather a clinical sign [8], and the pathogenesis is multifactorial, although histology shows a disruption of the mucosal integrity in all these cases [9]. Basically, there are three accepted theories [9, 10]: First, the gas pockets produced in pneumatosis are of bacterial origin (bacterial theory), especially in systemic infection [11]. Second, the gas is pushed into the bowel wall because of direct trauma of increased pressure (mechanical theory), which occurs in case of extreme luminal distension [12], or after previous interventions, like colonoscopy or transplantation [10, 13–16]. Third, the mucosal disruption is the primary causative factor, so that bacteria or air bubbles easily penetrate into the bowel wall. The latter is seen with bowel ischemia, inflammation, and previous chemotherapy for cancer or steroid treatment [17, 18].

Finally, PI can also occur with chronic obstructive pulmonary disease, or it may result from artificial ventilation with positive end-expiratory pressure, thus representing an incidental finding on MDCT without any need for treatment [1, 19, 20]. This so-called “pulmonary theory” has been explained by disruption of alveoli, followed by extension of gas through the mediastinum along the tissue planes and through the perivascular spaces into the bowel wall; however, definite proof is lacking [5, 9].

PI and PMVG are today increasingly detected, mainly because of the better sensitivity and the steadily growing use of MDCT in the emergency departments [2, 6, 21]. We had seen a rising number of patients in our emergency department in whom PI was incidentally detected and then turned out to be unrelated to intestinal ischemia. Thus, we were wondering if the clinical significance of PI should be revised.

By reviewing a large consecutive series of emergency patients, we aimed at investigating the exact clinical significance of PI in these acute situations. Furthermore, our goal was to investigate MDCT-findings, concomitant clinical and/or laboratory parameters that would help to distinguish emergency patients with PI due to underlying intestinal ischemia from those with PI secondary to non-life-threatening aetiologies. We especially wanted to know the prognostic value of associated PMVG, whether it truly deteriorated the patients’ outcome or occurred only as incidental sign [7, 22, 23].

## Materials and methods

This single-centre retrospective study was approved by our institutional ethics committee. Patients’ active consent was waived.

### Patients

We deliberately limited our study population to cases investigated in our radiological emergency department, thus including new, acutely admitted patients and in-patients with acute complications of their underlying disease. Therefore, each MDCT examination had been requested and performed in emergency. After entering the keyword “pneumatosis intestinalis” in our comprehensive electronic database of examination reports (search period from April 2006 to May 2014) we retrieved 187 patients in whom PI had been reported. Nine patients were excluded for being under 18 years old. Our study population was thus composed of 178 patients.

### Technical parameters

During the considered period (April 2006 to May 2014) all the MDCT examinations were performed on a 64-detector row CT machine (Lightspeed VCT; 64 Pro, GE Healthcare; Milwaukee, Wisconsin, USA). The imaging protocol included the whole abdomen and pelvis (diaphragm to pubic symphysis, 120 kV, 300-400 mA, table speed 55 m per rotation (0.8 s), pitch 1.375). The number of acquired abdominal passages (native, arterial and/or portal) depended on the individual clinical indication of the examination and on the patient’s individual contraindications (previous contrast medium reaction, renal failure). After a non-enhanced phase (2.5/2 mm reconstructed axial slices), we intravenously injected the iodinated contrast medium Accupaque® (Iohexol, 300 mgI/ml; GE Healthcare, volume in milliliters = body weight + 30 ml) at a flow rate of 4 ml/s, followed by an arterial phase (25 s, 1.25/1 mm reconstructed axial slices) and a venous phase (80 s, 2.5/2 mm reconstructed axial slices). Automatic tube current modulation in all three axes (SmartmA) was used.

### Image analysis

Two senior radiologists (S.S., M.O.T.) with 15 and 7 years of subspecialty expertise in abdominal imaging, respectively, jointly reviewed all the MDCT examinations on a picture archiving and communication system (PACS) workstation (Carestream Vue, version 11.4; Carestream Health, Rochester, NY, USA). They only knew the presence of PI in each patient, without being aware either of the clinical and histological context, or of the final outcome. They analysed all the radiological findings listed in Table 1, while reviewing the MDCT images in soft tissue and also lung window settings. Pneumatosis was confirmed, when intramural air was seen on the ventral and on the dependent aspect of the bowel wall [12, 24, 25]. Thus, they avoided false positive findings corresponding to trapped air bubbles located between the wall and the fluid-filled lumen of the bowel.

**Table 1:** Radiological and clinical findings, laboratory tests, anamnestic data and final diagnosis assessed in each patient with PI

Radiological findings	PI - Location	Stomach
		Small bowel
		Colon
	PI - Extension	Segmental
		Regional
		Extensive
		Diffuse
	PMVG – Distribution [1]	Mesenteric arcade veins
		Segmental veins
		Superior mesenteric vein
		Extrahepatic portal vein
		Intrahepatic portal veins
	Bowel	Wall thickening
		Mural contrast hyperenhancement
		No mural contrast enhancement
		Luminal dilatation
	Vessels	Arterial thrombosis/embolus
		Venous thrombosis
		Calcified atherosclerosis
	Abdominal cavity	Mesenteric fat stranding
		Peritoneal free fluid
		Pneumoperitoneum
Clinical findings	Abdominal pain	
	Emesis/vomiting	
	Diarrhoea	
	Peritonism	
	Septic shock	
Laboratory tests	Serum lactate (>2.4 mmol/l)	
	WBC (>12 c/mm^3^)	
	Arterial pH (<7.34, >7.45)	
	BUN (>7.7 mmol/l)	
Anamnestic data	Cardiovascular risk factors	Coronary atherosclerotic disease
		Peripheral vascular disease
		Arterial hypertension
		Smoking
		Hyperlipidaemia
		Diabetes mellitus
		Obesity
	Previous surgery (<21 days)	Cardiovascular (thorax/abdomen)
		Thoracic (non cardiovascular)
		Abdominal (non vascular)
	Previous trauma/intervention (<21 days)	Endoscopic procedures (ERCP, colonoscopy, gastric dilatation)
		TACE
		Organ transplantation [13]
	Corticoid treatment	
Treatment	Conservative	
	Surgery	Exploratory
		Curative
Histopathology	Not done	
	No abnormality	
	Ischemia	
	Infection	
	Inflammation	
Final diagnosis	• Ischemia	With/without vascular occlusion
	• Mechanical, obstructive bowel dilatation	Cancer, adhesions
	• Paralytic, non-obstructive bowel dilatation	Metabolic origin, Pseudoobstruction
	• Infection	Bacterial peritonitis, septic shock, Clostridium difficile colitis, cholecystitis, neutropenic enterocolitis, infected ventriculoperitoneal shunt
	• Inflammation	Crohn’s disease, post radiation enteritis, perforated diverticulitis
	• Systemic disease	Connective tissue disease
	• Pulmonary disease	COPD, asthma, emphysema, fibrosis
	• Medications	Corticosteroids,chemotherapy, lactulose
	• Trauma/iatrogenic	Organ transplantation, colonoscopy…
	• Idiopathic, unknown	

PI – pneumatosis intestinalis, PMVG – portomesenteric venous gas, WBC – white blood cell count, BUN – blood urea nitrogen, TACE – transhepatic arterial chemoembolisation, LED – lupus erythematodes disseminatus, COPD – Chronic obstructive pulmonary disease

Location (stomach, small bowel, colon) and extension of PI were evaluated, the latter differentiated as segmental (0-5 cm of bowel wall length involved), regional (>5 cm of bowel wall length, but >50 % of the whole organ wall length), extensive (≥50 % of the organ wall length involved) or diffuse (involving the whole organ).

The other assessed bowel wall MDCT findings were pathological wall thickening (>3 mm, provided that bowel loops were not collapsed), pathological contrast enhancement of the bowel mucosa and decrease of the physiological, subtle contrast enhancement of the bowel mucosa [10, 26]. The small bowel was considered dilated, whenever the lumen measured >3 cm in diameter. The colon was considered dilated whenever the lumen measured >6 cm in diameter with the exception of the caecum, for which a lumen of >8 cm was considered dilated [12, 27].

The analysis of the bowel wall and the evaluation of the vascular lumen (thrombosis, embolus) were only feasible when IV iodinated contrast medium had been given during MDCT.

For each patient, the two radiologists jointly differentiated three degrees (subtle, moderate, and severe) of calcified atherosclerosis by evaluating the extension of the mural calcifications of the aorta and the other abdominal arteries.

The presence and extension of PMVG were classified, as described by Heye et al. [1], into venous arcades, segmental mesenteric veins, the superior mesenteric vein, the extrahepatic portal vein, and the intrahepatic portal vein.

After reading the MDCT images, the two radiologists reviewed each electronic patient file in order to collect all the clinical, laboratory, and histological findings shown in Table 1. Only laboratory tests that had been collected on the same day as the MDCT examination were considered. In case of several results on the same day, the one closest to the time of the MDCT acquisition was chosen. For the anamnestic data, any previous surgery and other interventions that were done 21 or fewer days before the MDCT examination were considered, since these may have been related to the PI seen on the images [17, 18, 20].

Our reference standard was the final diagnosis that was based on the patient’s histological result, if available, together with the medical investigations that were performed and the discharge letter. Thus, the final diagnosis resulted from a combination of histological and clinical findings. In all cases, the final diagnosis that was considered in our study was the diagnosis obtained from the medical charts based on multiple investigations and reported on the discharge letter.

According to Taourel et al., the purely mechanical origin of PI was diagnosed when MDCT showed mechanical bowel obstruction with PI, either with histology excluding bowel wall ischemia or with a follow-up MDCT showing spontaneous regression of PI including the luminal dilatation (after successful treatment) [12]. We diagnosed non-obstructive bowel dilatation and PI of metabolic origin [28] whenever histology excluded ischemia or conservative treatment was successful, with spontaneous regression of the radiological signs. Epidemiological findings and the final outcome were also assessed in each patient, especially in cases of death during the same hospital stay.

### Statistical analysis

Statistical analysis was performed with the commercially available software R (R Core Team (2013). R: A language and environment for statistical computing. R Foundation for Statistical Computing, Vienna, Austria. URL http://www.R-project.org/.)). Data are presented as number and relative percentages. Categorical variables were compared with the Chi-square test, for continuous variables the Student’s test or the analysis of variance (ANOVA) were applied. Statistical difference was considered significant for a *p*-value < 0.05. For the problem of multiple testing, the *p*-values were adjusted using the False Discovery Rate (FDR) methods [29].

## Results

We had to exclude 29 patients during our image analysis, because PI could not be confirmed by the two readers. Thus, our final study population included 149 patients (53 women, 96 men; mean age 64 years, range 21-95).

Intravenously given iodinated contrast-enhanced MDCT-acquisition had been performed in 103 patients (69.2 %), while 46 (30.8 %) patients underwent non-enhanced MDCT only. Among the 103 patients with IV contrast medium injection, 38 patients, in whom bowel ischemia was the primary clinical suspicion, had arterial and venous acquisition, and 64 patients with unspecific clinical working diagnoses had venous acquisition only. One patient, investigated for suspicion of aortic disease, had arterial acquisition only.

Pneumatosis intestinalis was located in the gastric wall in four patients (2.7 %), in the small bowel wall in 68 (45.6 %), and the colonic wall in 96 (64.4 %) patients. Among them, PI was observed in one (0.7 %) patient in the gastric and small bowel walls simultaneously, and in 18 (12.1 %) patients in the small and large bowel walls simultaneously.

**Fig. 1:**
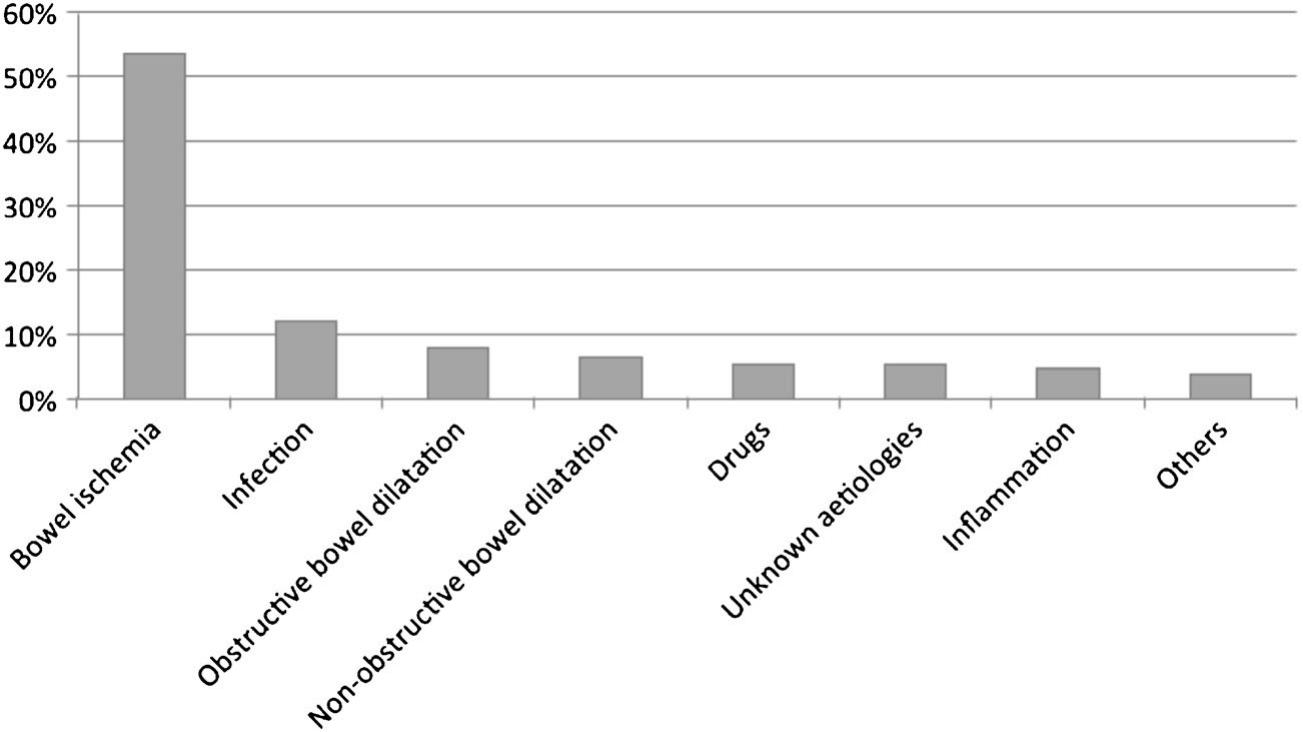
Overview of the different aetiologies (in percentages) of pneumatosis intestinalis presented by our study population

**Fig. 2:**
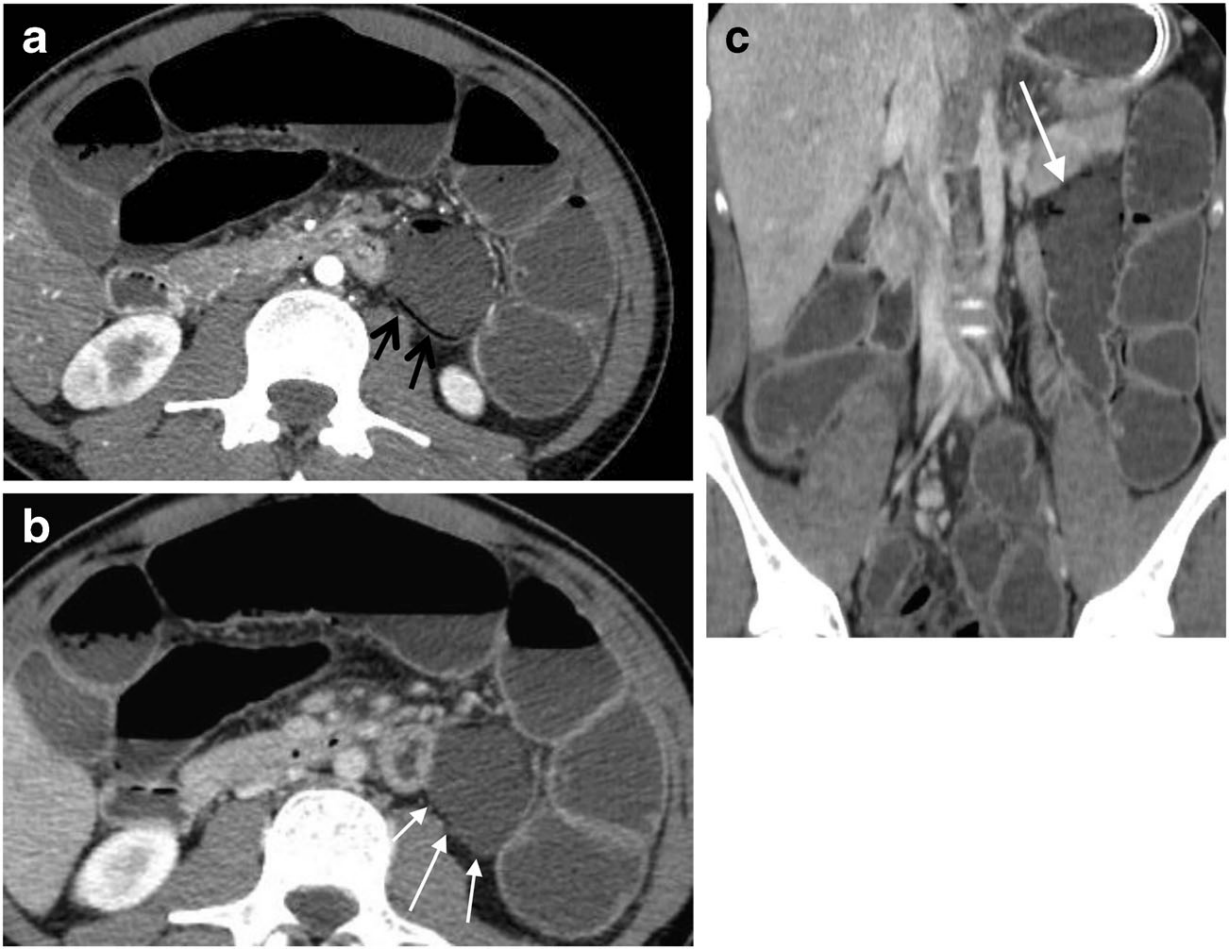
A 19-year-old patient known for ulcerative colitis developed septic shock 3 days after confection of a J-Pouch. Axial (**a**, **b**) and coronal (**c**) MDCT images reveal PI (**a**, *black arrows*) of a jejunal loop associated with absent mural contrast enhancement (**b**–**c**, *white arrows*), thus clearly indicating acute ischemia

**Fig. 3:**
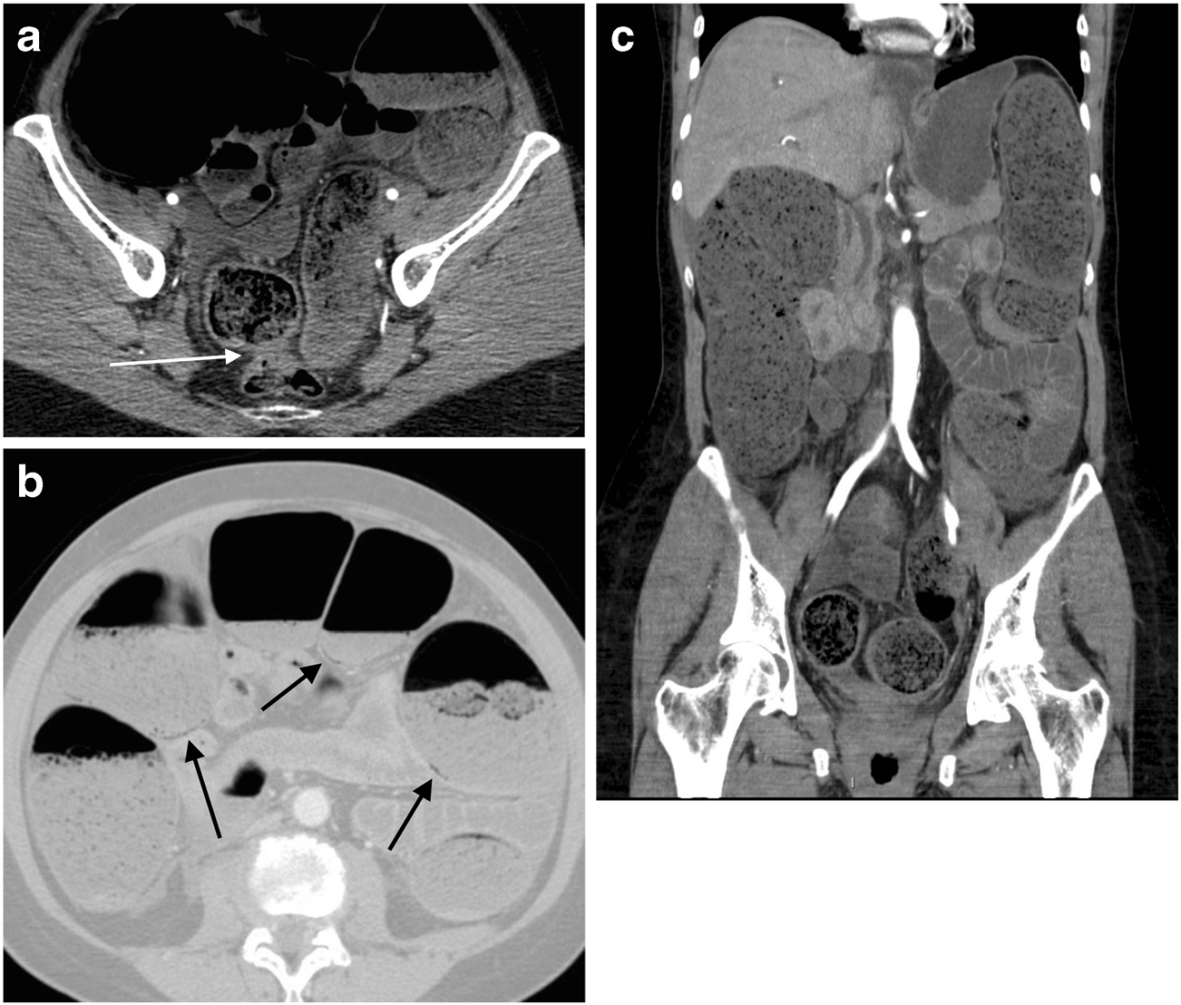
A 63-year-old woman presented in emergency with acute abdominal pain and elevated serum lactate (3.1 mmol/l). Axial (**a**, **b**) and coronal (**c**) MDCT images reveal a recto-sigmoid cancer (**a**, *white arrow*) causing mechanical obstruction with proximal luminal dilatation, fecal stasis (**b**–**c**) and PI (**b**, *black arrows*). Total colectomy was immediately performed, resecting both the tumour and the whole proximal colon because of ischemic necrosis of the latter

**Fig. 4:**
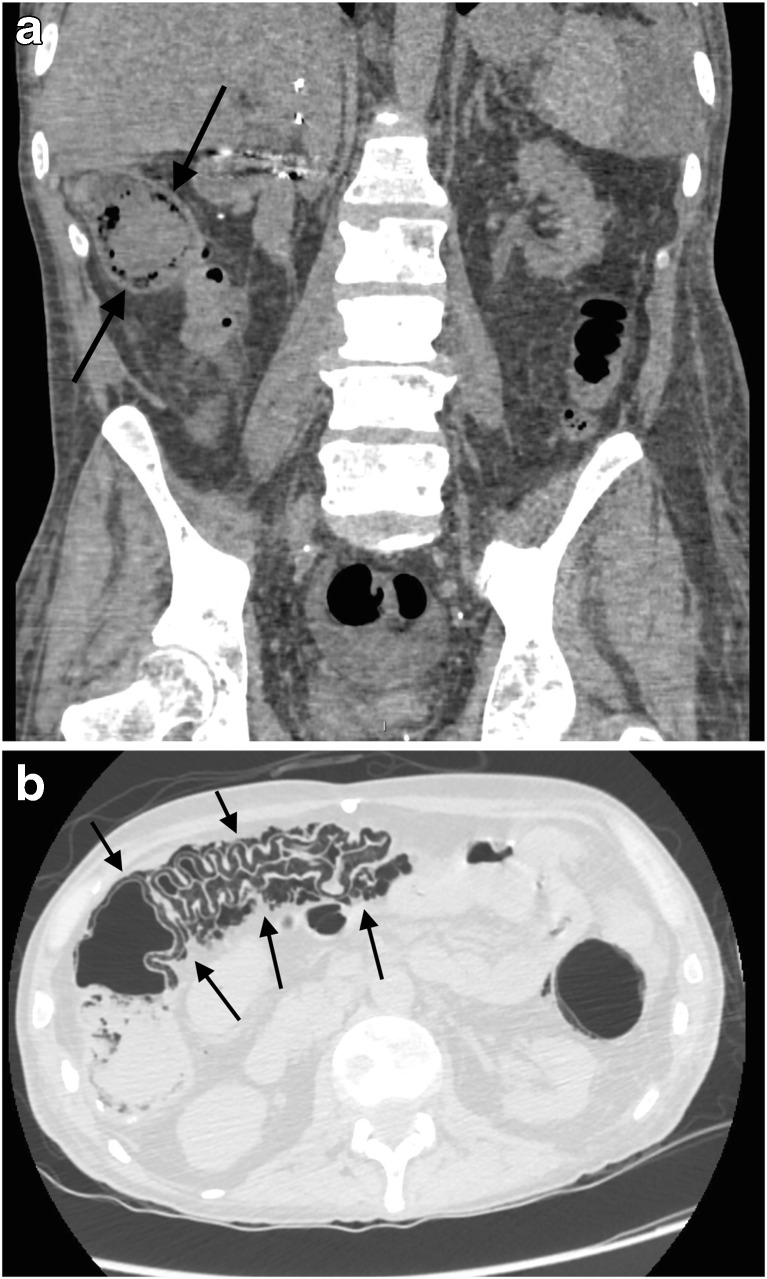
**a**–**b** A 51-year-old man known for multimetastatic ileal neuroendocrine tumour, and previously treated with surgery, hepatic radioembolisation, and systemic radiotherapy (Dotatoc®), presented with severe abdominal pain and peritonism. Lab tests, including lactate, were normal. Coronal (**a**) and axial (**b**) non-enhanced MDCT-images reveal extensive colonic PI (*black arrows*), confirmed by immediate laparotomy, but the colon was viable. The aetiology of PI remained unknown

The most frequent causes of PI are shown in Fig. 1. Bowel ischemia was the most frequent aetiology (*n* = 80, 53.7 %, Fig. 2), followed by infection (*n* = 18, 12.1 %), obstructive (*n* = 12, 8.1 %, Fig. 3) and non-obstructive (*n* = 10, 6.7 %) bowel dilatation, drugs (*n* = 8, 5.4 %), unknown aetiologies (*n* = 8, 5.4 %, Fig. 4), inflammation (*n* = 7, 4.7 %), and others (*n* = 6, 4 %). Histological proof of diagnosis was available in 91 patients (61.1 %).

The localization of PI caused by ischemia was the stomach in two patients (50 % of the stomach PI), the small bowel in 42 patients (61.8 % of the small bowel PI), and the colon in 48 patients (50 % of the colon PI). One of the two patients with ischemic gastric PI also had small bowel involvement and in 11 of the patients with ischemic PI the small bowel and the colon were involved simultaneously.

Table 2 shows the statistical relations between PI caused by bowel ischemia and the evaluated findings. Neither the anatomical location of PI (involvement of the small (*p* = 0.19) or large bowel (*p* = 0.46) or both together nor the length of intestinal involvement by PI (*p* = 0.92) significantly correlated with underlying ischemia. PMVG occurring together with PI was significantly associated with underlying ischemia (*n* = 48, *p* = 0.009). Three of the five evaluated sites of anatomical distribution of PMVG also significantly indicated an ischemic origin of PI, that is, PMVG located in the segmental veins (*n* = 23, *p* = 0.027), in the extrahepatic portal vein (*n* = 14, *p* = 0.027), and in the intrahepatic portal veins (*n* = 35, *p* = 0.003). A statistically significant tendency was shown for the arcade veins (*n* = 36, *p* = 0.06). The only exception was PMVG located in the superior mesenteric vein (*n* = 9, *p* = 0.78).

**Table 2:** Relations between PI caused by bowel ischemia and the evaluated radiological, clinical, and laboratory findings

Radiological findings	PI – location	
	Stomach	χ^2^(1) = 0.02; p = 0.881/p = 0.95
	Small bowel	χ^2^(1) = 3.28;p = 0.070/p = 0.189
	Colon	χ^2^(1) = 1.48; p = 0.223/p = 0.46
	PI - extension	χ^2^(3) = 0.91; p = 0.824/p = 0.92
	PMVG - presence	χ^2^(1) = 10.75;p = 0.001*/p = 0.009*
	PMVG – distribution	
	Mesenteric arcade veins	χ^2^(1) = 5.73; p = 0.016*/p = 0.06
	Segmental veins	χ^2^(1) = 7.97; p = 0.005*/p = 0.027*
	Superior mesenteric vein	χ^2^(1) = 0.45; p = 0.501/p = 0.78
	Extrahepatic portal vein	χ^2^(1) = 8.24; p = 0.004*/p = 0.027*
	Intrahepatic portal veins	χ^2^(1) = 13.42;p < 0.001*/p = 0.003*
	Bowel	
	Wall thickening	χ^2^(1) = 0.26; p = 0.610/p = 0.78
	Mural contrast hyperenhancement	χ^2^(1) = 0.32; p = 0.569/p = 0.78
	Decreased mural contrast enhancement	χ^2^(1) = 20.06; p < 0.001*/p < 0.001*
	Luminal dilatation	χ^2^(1) = 0.34; p = 0.558/p = 0.784
	Abdominal cavity	
	Mesenteric fat stranding	χ^2^(1) = 2.69; p = 0.101/p = 0.22
	Peritoneal free fluid	χ^2^(1) = 0.00; p = 0.987/p = 0.99
	Pneumoperitoneum	χ^2^(1) = 0.11; p = 0.743/p = 0.911
	Atherosclerosis	χ^2^(3) = 9.42; p = 0.024*/p = 0.08
Clinical findings	Abdominal pain	χ^2^(1) = 5.26*10^-6^; p = 0.998/p = 0.99
	Emesis/vomiting	χ2(1) = 0.41; p = 0.521/p = 0.78
	Diarrhoea	χ2(1) = 0.02; p = 0.335/p = 0.60
	Peritonism	χ^2^(1) = 2.81; p = 0.093/p = 0.22
	Septic shock	χ^2^(1) = 1.15; p = 0.282/p = 0.54
Laboratory tests	Serum lactate (>2.4 mmol/l)	t(104) = |0.7|; p = 0.483/p = 0.78
	WBC (>12 c/mm^3^)	t(145) = |2.67|; p = 0.008*/p = 0.03*
	Arterial pH (<7.34)	t(103) = |0.70|; p = 0.051/p = 0.15
	BUN (>7.7 mmol/l)	t(95) = |0.27|; p = 0.785/p = 0.92

The correspondent p-values are shown before (first p-value) and after (second p-value) adjustment using the False Discovery Rate method (FDR) [29]

Significant statistical differences are indicated with an asterisk (*)

Among all the other evaluated radiological MDCT wall features, only the feature “decreased mural contrast-enhancement” significantly correlated with ischemia (*n* = 23, *p* < 0.001).

Arterial coeliac/mesenteric thrombosis or embolus was observed in 13 patients, among them 12 patients with bowel ischemia and one with obstructive bowel dilatation.

Venous mesenteric thrombosis was observed in one patient. This patient, known for a long history of liver cirrhosis and ascites, developed a thrombosis of the superior mesenteric vein with associated PI of the small bowel and the caecum in the context of proven bacterial peritonitis (*Enterococcus faecium* and *Staphylococcus aureus*).

The degree of calcified atherosclerosis, as jointly evaluated by the two radiologists on the MDCT images, showed a statistical tendency to be significantly associated with underlying bowel ischemia (*p* = 0.08), unlike the anamnestic cardiovascular risk factors registered from the patients’ records.

None of the clinical findings present at the time of MDCT acquisition significantly correlated with an ischemic origin of PI (Table 2).

As for the laboratory tests, values of serum lactate (mean 3.6 mmol/L, range 0.3-24.0 mmol/L) were available in 106 patients (71.1 %), values of pH (mean 7.33, range 6.78-7.55) in 105 patients (70.5 %), values of blood urea (mean 17 mmol/L, range 3.3-63.0 mmol/L) in 97 patients (65.1 %), and values of the white blood count (WBC) (mean 14.1 G/L, range 0.2-54.7 G/L) in 147 patients (98.7 %). Only the WBC (*p* = 0.03) was statistically significantly associated with underlying bowel ischemia, unlike the other laboratory tests, namely serum lactate, pH and the blood urea (Table 2).

**Fig. 5:**
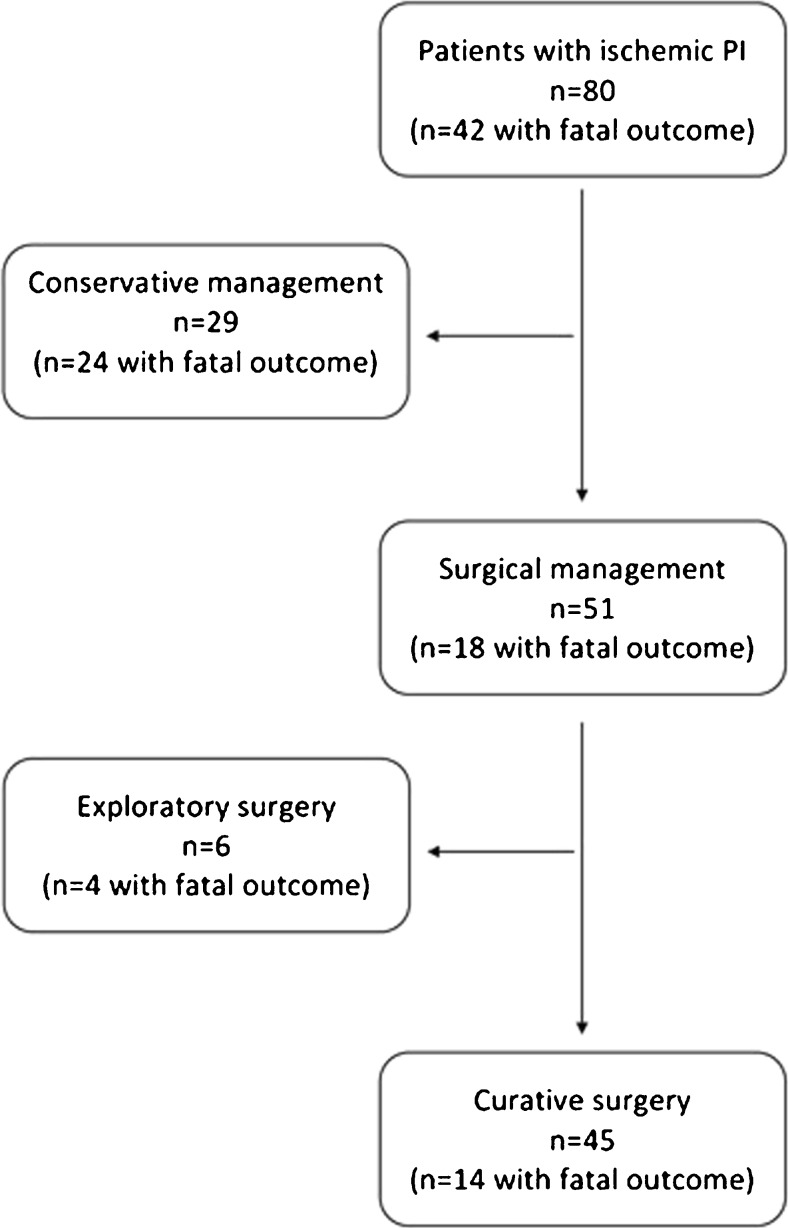
Flow chart giving an overview of the chosen treatment in patients with ischemic PI

The choice of treatment in patients with ischemic PI is shown in a flow chart (Fig. 5). Fifty-one of 80 patients with ischemic PI underwent surgery (63.8 %). Twenty-nine patients with ischemic PI were treated conservatively. One patient had ischemic colitis occurring after interventional embolisation of the right colic arteries because of acute colonic bleeding. He was treated conservatively with success. The other 28 non-operated patients had a fatal outcome the same day or following days after imaging.

When taking into account the whole study population, the group of patients undergoing surgery had a significant higher likelihood to have an ischemic origin of PI (*p* < 0.001) than those treated conservatively. Even the likelihood to undergo curative surgery (*n* = 45, *p* = 0.012), instead of exploratory (*n* = 6) only, was significantly higher in patients with ischemic PI.

Overall mortality of the study population was 41.6 % (*n* = 62), and was significantly related to intestinal ischemia (*p* = 0.003). Mortality of patients with ischemic PI was 52.5 %. The median time interval between PI seen on MDCT and fatal outcome was 2 days (range 0-56, mean 8.8). The patients with PI caused by ischemia were significantly older (67.9 years) than the patients with PI resulting from other causes (59.4 years, *p* < 0.001). However, fatal outcome did not depend on age in the group with ischemic PI. In these patients, mortality was not significantly related to the presence of PMVG (*p* = 0.701) either.

## Discussion

Our study including 149 consecutive emergency patients confirmed that PI may be caused by various disorders. However, unlike our initial hypothesis that, today, non-life-threatening aetiologies are commonly associated with PI, possibly more commonly than bowel ischemia, the latter turned out to represent 53.7 % of the aetiologies of PI. Thus, in more than half of our patients, PI indicated either primary (i.e., occlusive or non-occlusive vascular origin) or secondary ischemia (i.e., due to bowel obstruction). Each of the other various aetiologies of PI were far less frequent, among them infection being the most common cause, occurring in 18 patients (12.1 %).

Furthermore, we initially thought that PI was not, on its own, a good indicator of underlying ischemia. Our findings confirm this hypothesis, but further reveal that when accompanied by PMVG, PI significantly indicates underlying intestinal ischemia. Indeed, among the 69 patients with PI and PMVG in our study, 48 (69.6 %) had bowel ischemia. While Heye et al. [1] reported that PMVG located in the arcade vessels was the best indicator for ischemia compared to other, more proximal, sites of PMVG, our study is the first to show a statistically significant correlation for any possible location except for gas located in the superior mesenteric vein. This latter result is likely a random finding and clinically insignificant, since PI and PMVG represent progressive steps in a single pathophysiological process. Gas progressively ascends from the bowel wall to the liver, extending through the arcade veins, the segmental veins, the superior mesenteric vein, and finally into the extra- and intrahepatic portal veins [1, 7, 19, 30]. Nevertheless, although significantly correlated with ischemia in our study, PMVG may result from various other conditions, as reported by Hussain et al. [7], namely the increase of luminal pressure in case of obstructive and non-obstructive bowel dilatation as well as bacterial contamination of the portomesenteric venous system from the digestive tract. However, other MDCT findings, (most helpfully, decreased bowel wall enhancement) may give clues of the underlying disease.

Decreased mural contrast-enhancement was the only other radiological bowel finding that very strongly correlated with an ischemic origin of PI. To our knowledge, we are the first working group to report this statistical association, since these two findings have not been previously evaluated together [17, 19]. Recently, Millet et al. have stressed the clinical importance of reduced bowel wall enhancement in the context of bowel ischemia caused by small bowel obstruction with strangulation, but without associating PI to their analysis [31]. However, in some acute situations, such as in case of important bowel dilatation, decreased bowel wall enhancement may be difficult to assess, since the wall is then very thin and distinguishing between PI of mechanical and ischemic origins becomes difficult (Fig. 3).

None of our evaluated clinical signs significantly correlated with ischemic PI, in contradistinction to other authors reporting a significant association between abdominal pain [6] or peritoneal signs [19] and an ischemic PI/PMVG. We explain this difference by the fact that some of our patients were intubated and/or sedated, thus not able to communicate the symptoms or to be accurately examined clinically.

The only laboratory test that significantly correlated with ischemia in our study, was the white blood count, in agreement with Greenstein et al. [5] and Hussain et al. [7], but in contradistinction to Wayne et al. [6]. The latter two authors, however, found a significant association between acute mesenteric ischemia and an elevated lactate level [6, 7], which we could not confirm. On the other hand, in our study the mean lactate level was increased (3.7 mmol/l), with a wide range of 0.3-24.0 mmol/L. This may be explained by other important, sometimes acute comorbidities present in many of our patients. Indeed, according to Demir et al., the serum lactate is rather an unspecific marker of tissue hypoperfusion than a specific marker of acute bowel ischemia, whereas no single serum marker shows a sufficient sensitivity and specificity for the reliable diagnosis of acute bowel ischemia [32].

Hani et al. [19] reported a significant association between high blood urea nitrogen (BUN) and ischemic PI, unlike our results. We could only show a clearly elevated mean BUN, probably reflecting the bad general condition and the critical situation of our patients at the time of the MDCT examination.

Cardiovascular risk factors (noted in the patients’ files) did not significantly correlate with the ischemic PI, unlike the results reported by Wayne et al. [6]. The degree of calcified atherosclerosis, as scored in consensus by the two radiologists during our image analysis, showed a tendency to be significantly associated with ischemic PI. This stresses the importance of an accurate vessel analysis, whenever acute intestinal ischemia is suspected clinically.

The percentage (53.7 %) of ischemic PI observed in our study group nicely agrees with the results reported by Lassandro et al., who retrospectively evaluated the diagnostic and prognostic value of PI in 102 patients [33]. When PMVG occurred together with PI, the ischemic origin was even more likely, also in agreement with our and other [34] results. Unfortunately, Lassandro et al. did not take into account any radiological or clinical associated findings [33].

Some authors distinguish between a bubbly and a linear pattern of PI seen on MDCT [12, 21, 24]. They advocated a benign origin in case of a bubbly or cystic PI and a malignant (thus life-threatening) aetiology in case of a linear or curvilinear pattern of PI. However, the authors admitted that none of these patterns was pathognomonic of one or the other category. Therefore, we did not consider the appearance of PI, either bubbly or linear, in our study.

There were several limitations to this study. First, the main limitation is that histopathological proof was available in 61 % of our patients only (*n* = 91), since it was impossible to be obtained for all of them. However, it is unrealistic to have a histological proof for all the patients as not all of them were suitable candidates for surgical therapy depending on their medical condition. We tried to compensate for this by thoroughly reviewing the patients’ electronic files and taking into account as many anamnestic, clinical, and laboratory findings as possible. Thus, our reference standard was the final diagnosis that was based on the patient’s histological result, if available, together with the medical investigations that were performed and the discharge letter. Second, the retrospective character of our study may, by definition, include bias. Third, 46 of the 149 patients (30.8 %) included in this study underwent unenhanced MDCT, thus rendering impossible the evaluation of bowel wall enhancement and vascular findings.

In conclusion, our retrospective analysis of 149 emergency patients with PI suggests that bowel ischemia remains the most common cause. Among all the considered clinical signs, laboratory investigations, and MDCT features, concomitant PMVG, decreased bowel wall enhancement, and severe atherosclerosis highly significantly correlate with an ischemic origin of PI. Given the significant relation between ischemic PI and mortality as well as the absence of significant relation between ischemic PI and clinical findings or most laboratory tests, diagnosing the aetiology of PI remains a challenging situation for both the clinician and the radiologist that must not be trivialized.
